# Association Between Information and Communication Technologies (ICTs) and Women’s Attitudes Toward Intimate Partner Violence: Evidence from Bangladesh Demographic and Health Survey 2017–2018

**DOI:** 10.3390/bs14111012

**Published:** 2024-10-31

**Authors:** Ashim Kumar Nandi, Bijoya Sarkar, Md. Nazmul Huda, Navira Chandio, Kh. Shafiur Rahaman, Amit Arora

**Affiliations:** 1 Department of Sociology, University of Barishal, Barishal 8254, Bangladesh; univbu.bijoya.sm@gmail.com; 2 Infant, Child and Adolescent Mental Health Service, South-Western Sydney Local Health District, Liverpool, NSW 2170, Australia; m.huda@unsw.edu.au; 3 Discipline of Psychiatry and Mental Health, University of New South Wales (UNSW), Sydney, NSW 2052, Australia; 4 Ingham Institute for Applied Medical Research, Liverpool, NSW 2170, Australia; 5 School of Health Sciences, Western Sydney University, Campbelltown Campus, Locked Bag 1797, Penrith, NSW 2751, Australia; 18154463@student.westernsydney.edu.au (N.C.); 22108248@student.westernsydney.edu.au (K.S.R.); a.arora@westernsydney.edu.au (A.A.); 6 Health Equity Laboratory, Campbelltown, NSW 2560, Australia; 7 Translational Health Research Institute, Western Sydney University, Campbelltown Campus, Locked Bag 1797, Penrith, NSW 2751, Australia; 8 Oral Health Services, Sydney Local Health District and Sydney Dental Hospital, NSW Health, Surry Hills, NSW 2010, Australia; 9 Discipline of Child and Adolescent Health, Sydney Medical School, Faculty of Medicine and Health, The University of Sydney, Westmead, NSW 2145, Australia

**Keywords:** intimate partner violence, information and communication technologies, women’s health, public health, Bangladesh, BDHS

## Abstract

Many women justify intimate partner violence (IPV), resulting in adverse health outcomes. This study investigates the relationship between household ownership of information and communication technologies (ICTs), along with the frequency of listening to the radio and watching television with women’s attitudes towards IPV in Bangladesh. The cross-sectional study analyzed a weighted sample of 20,032 women and used a multivariable logistic regression analysis to determine the association between predictor variables and outcome variables. The results showed that 19.47% of women justified wife beating for at least one reason. We found that household ownership of computers (AOR = 0.73 [95% CI = 0.57, 0.95]), women in households with three ICTs (AOR = 0.67 [95% CI = 0.47, 0.96]), and women who watched television at least once a week (AOR = 0.85 [95% CI = 0.74, 0.97]) were associated with decreased odds of justifying wife beating for at least one reason after adjustment for the frequency of reading newspaper or magazine, age, wealth, education, religion, and type of place of residence. The study suggests that not all ICTs affect women’s attitudes toward IPV equally. Computers and television were more influential than other ICTs. This finding suggests that awareness-building and educational programs targeted towards women via computer and television may deliver better outcomes about gender norms.

## 1. Introduction

Intimate partner violence (IPV) includes actions carried out by a current or ex-intimate partner that result in physical, sexual, or psychological harm, which includes acts of physical aggression, sexual manipulation, psychological mistreatment, and controlling behaviors [1]. IPV is a significant public health problem [2]. Analyzing data from 6.1 billion people from 133 countries, the World Health Organization (WHO, 2014) reported that one in three women experienced physical or sexual violence from their intimate partner at least once in their lifetime. The prevalence of IPV was reported to be 29.8% in the WHO region of the Americas, 23.2% in high-income countries, 25.4% in the WHO European region, 37.0% in the WHO Eastern Mediterranean region, 36.6% in the WHO African region, 24.6% in the Western Pacific region, and 37.7% in the South-East Asia region [3]. Globally, around 27.0% of women aged 15–49 years are the victims of IPV [2]. In Bangladesh, the context of this research, approximately 72.6% of married women experienced one or more instances of violence from their spouses at least once in their lifetime [4].

IPV is a significant risk factor for HIV, other sexually transmitted diseases, unwanted pregnancies, and reproductive health problems [5]. Women with intimate partner violence have a 16.0% higher risk of having a low-birth-weight baby and are more than twice as likely to have an induced abortion [5]. In addition, in certain regions, they are 1.5 times more likely to acquire HIV and 1.6 times more likely to have other sexually transmitted diseases such as syphilis [5]. Furthermore, women who have been subjected to IPV have a nearly two-fold higher risk of developing an alcohol use disorder, are twice as likely to experience depression, and have an elevated risk of attempting suicide compared to women who have not experienced partner violence [3]. Women who have experienced IPV are more likely to suffer from chronic pain, respiratory diseases, gynecological symptoms, sexually transmitted infections, HIV [6], depression, post-traumatic stress disorder, anxiety, and suicide [7,8]. In addition, some meta-analyses show that individuals who have experienced IPV are three to five times more likely to report symptoms of depression, anxiety, and post-traumatic stress disorder (PTSD) [9].

Multiple studies in low- and middle-income countries (LMICs) have shown that specific forms of IPV are culturally accepted, particularly by the victims themselves [10,11,12,13,14]. Gunarathne et al. [15], in a systematic review of the LMICs context, found that the adherence to gender norms contributes to the high occurrence of IPV in LMICs. In another systematic review focused on LMICs, Ghoshal et al. [16] revealed that when any spouse justifies wife beating, it leads to an increase in the risks of IPV. Previous research emphasized the significance of adopting an unsupportive stance towards IPV as a means of decreasing the overall occurrence of IPV incidents [17]. Women who had tolerant attitudes towards wife beating were more likely to experience IPV in 13 out of 15 research locations, as reported by the WHO [18]. Rahman et al. [19] found that Bangladeshi women who held a negative attitude towards violence were 39% less likely to report experiencing IPV in their lifetime. Therefore, identifying factors enhancing women’s awareness of IPV is critical to addressing this public health problem in Bangladesh.

ICTs can enhance women’s awareness of their health and well-being rights. Technology-driven therapies are increasingly becoming popular for providing support to survivors of IPV and are offered in various forms such as smartphone apps, web and phone-based decision assistance tools, chatbots, text messages, online support groups, social media platforms, and telehealth services [20,21,22,23]. The growing proficiency of patients in using technology, device ownership, and advancements in device capabilities has led to a greater dependence on technology for providing interventions for IPV survivors, allowing for round-the-clock, secure, and confidential services and resources [24,25,26]. In the last three decades, digital therapies of different complexities have been developed and adjusted to assist in the health and overall wellbeing of survivors of IPV. These interventions have become an essential form of support for IPV survivors [27]. Some studies show that survivors of IPV are receptive to trauma-informed technology-based interventions [22,26,28]. Self-administered computer screening is more effective than face-to-face screening in identifying cases of IPV by 37% and surpassing paper and pencil screeners by 23% [29]. Digital IPV interventions are designed to address limitations of traditional methods, such as high costs, lack of confidentiality, inadequately trained healthcare providers, limited access due to geographical barriers, social stigma, and variations in socio-geographical factors [22,25,28,30,31,32]. A review by Fu et al. [33] reported that digital psychological therapies effectively addressed IPV-related mental health outcomes for adults in LMICs. Furthermore, EI Morr and Layal [34], through a systematic review, evaluated the effectiveness of ICT-based IPV interventions. They [34] found that ICT-based interventions were effective in IPV screening, disclosure, and prevention.

Previous literature suggests that ICTs and IPV are likely to be associated with each other [27,35]. Cardoso and Sorenson [35] found a positive correlation between ICTs in a household and a lower risk of women’s acceptance of IPV, such as wife beating. A systematic review by Emezue et al. [27] found that technology-based interventions significantly reduced depression, anxiety, and physical violence victimization among female IPV survivors at 0–3 months. However, they added that no digital interventions significantly reduced PTSD or sexual violence victimization among female IPV survivors. Another study found that 62.0% of women who used ICT were less likely to report physical IPV, 76.0% were less likely to report IPV with damage, and 78.0% were less likely to report serious sexual IPV [36]. Braithwaite et al. [37] found a significant decrease in physical aggression incidents among both genders at a 1-year follow-up. They added that the increased use of ICTs in LMICs suggests the potential for ICT-based treatments. Many factors explain the acceptance of IPV. Earlier studies found that women’s acceptance of IPV is associated with women’s dependence on their husbands for financial support and social security [38], a lower level of educational attainment [39,40,41,42,43,44,45,46,47], a lack of household ownership of information communication technologies [35], a lack of household decision-making powers [48], and the influence of conservative religious customs, teachings, values, and belief systems [49,50,51,52,53].

Bangladesh is a society predominantly governed by men and follows a patriarchal system [54]. Bangladesh’s ICT sector is expected to grow, ranking 147 out of 176 on the International Telecommunication Union (ITU)’s ICT Development Index [55]. According to the Mobile Connectivity Index (MCI), Bangladesh has improved from 42 in 2017 to 51 in 2023 [56]. Moreover, being a middle-income country with a paucity of trained healthcare providers and limited access to IPV intervention due to geographical barriers, Bangladesh is a vital context to test the association between ICTs and IPV. Consequently, this study aims to examine the relationship between ownership and use of ICTs and women’s justifications for wife beating in Bangladesh. The specific research objective is to examine whether household ownership of mobile telephones, radios, televisions, and computers is associated with women’s justifications for wife beating in Bangladesh. Also, this study will investigate whether the frequency of listening to the radio and watching television is associated with women’s wife beating justifications.

## 2. Materials and Methods

### 2.1. Data Source

The 2017-18 Bangladesh Demographic and Health Survey (BDHS) was conducted by the National Institute of Population Research and Training (NIPORT) under the Medical Education and Family Welfare Division of Ministry of Health and Family Welfare. Mitra and Associates, a private research agency, was contracted to gather data from October 2017 to March 2018. The US Agency for International Development (USAID)/Bangladesh funded the survey, and ICF provided technical assistance through the DHS Program [57].

### 2.2. Sampling Design and Sample Size

The survey utilized a two-stage stratified random sampling approach to ensure that the sample represented the whole country. The survey utilized a sampling frame provided by the Bangladesh Bureau of Statistics (BBS), consisting of a list of enumeration areas (EAs) obtained from the 2011 Population and Housing Census of the People’s Republic of Bangladesh [58]. The survey’s primary sampling unit (PSU) was an EA with an average of approximately 120 households. During the initial phase, 675 EAs were chosen, with 250 in urban regions and 425 in rural areas. The selection was based on the proportional probability of the size of each EA. A systematic sample of approximately 30 households per EA was chosen for the second phase. Based on this design, 20,250 residential households were selected. The study has a total weighted sample size of 20,032. The specifics of the sampling process, data collection procedure, and questionnaire can be found in the 2017-18 BDHS final report [57].

### 2.3. Outcome Measure

The approval or justification of wife beating was the dependent variable. The justification for a husband to physically assault his wife was examined under five circumstances: if the wife burns the food; if the wife neglects the children; if the wife argues with the husband; if the wife goes out without telling the husband; and if the wife refuses to have sex with the husband. The questions were framed as either yes = 1 or no = 0. A comprehensive index was generated, including all the “yes” and “no” answers, and standardized on a scale of 0 to 5. Scores ranging from 1 to 5 were labeled as “approve”, and the score 0 was labeled as “not approve” [10,39,59]. A binary dummy variable was created, where a score of 0 implies that respondents disapprove of wife beating. In contrast, a score of 1 indicates that respondents approve of at least one of the five questions that justify wife beating.

### 2.4. Independent Variables

The predictor variable of interest consisted of household ownership (yes or no) of four types of ICTs (i.e., mobile telephones, radio, television, and computer) [35], the frequency of listening to the radio, and the frequency of watching television [39].

The covariates were the frequency of reading newspapers or magazines, age (categorized as age in five-year groups, such as 15–19, 20–24, 25–29, 30–34, 35–39, 40–44, and 45–49), wealth index combined (categorized as poorest, poorer, middle, richer, and richest), educational attainment (categorized as no education, incomplete primary, complete primary, incomplete secondary, complete secondary, and higher), religion (categorized as Islam, Hinduism, Buddhism, and Christianity), type of place of residence (categorized as urban, and rural), currently/formerly/never in union (categorized as currently in union/living with a man, and formerly in union/living with a man), and sex of household head (categorized as male and female) [10,35,39,40,60].

### 2.5. Statistical Analysis

Descriptive statistics were performed to calculate the number and frequencies in the univariable analysis. Binary logistic regression was performed to identify the factors associated with the justifications for the wife’s beating. Variables found significant in the bivariate analysis were considered for regression analysis. Multivariable logistic regression models were used to estimate the adjusted odds ratio (AOR) at 95% confidence intervals. Variance Inflation Factor (VIF) was used to check the multicollinearity (please see Table A1 in Appendix A). The VIF values (ranging between 1.00 and 3.52) are low for all variables in the model. As the values are below the suggested cut-offs, we do not have to worry about multicollinearity in our model. Stata version 18, a statistical software developed by StataCorp LP in College Station, TX, USA, was used for the analysis. In the analyses, we used Stata’s ‘svyset’ and ‘svy’: commands to modify the impact of this complex survey design.

## 3. Results

### 3.1. Univariable Analysis

Of the 20,376 ever-married reproductive-aged women between 15 and 49 years who were eligible for interviews (the study sample includes girls children between 15 and 18 years and women between 19 and 49 years. However, for an easier presentation, we used the word “women” to mean all the respondents aged 15–49 years), 20,127 were interviewed, providing a response rate of 99%. The women’s primary cause of non-response was their continual absence from home despite multiple visits. The response rates were similar based on whether they lived in urban or rural areas. The results of this study were obtained from 20,032 women participants. Table 1 shows the socio-demographic characteristics of the study participants. Of the 20,032 participants, 3901 women (19.47%) approved or justified wife hitting or beating by the husband for at least one of the five reasons, such as if the wife goes out without telling the husband, the wife neglects the children, the wife argues with the husband, the wife refuses to have sex with the husband, and the wife burns the food. Among the total 20,032 respondents, 19,391 women (96.8%) replied that their household had mobile telephones, which was the most common information and communication technology that a household had. The second common ICT was television; 10,106 women, or 50.42%, answered that their families had television. The least common ICT was radio. Of the participants, 1403 women (7.0%) and 208 women (1.04%) replied that their household had computers and radios, respectively.

Table 2 lists the socio-demographic factors and household ICTs ownership status that are known or assumed to be associated with the women’s justification of wife beatings by husbands for at least one reason. Women whose households had mobile telephones, televisions, and computers were less likely to justify wife beatings by their husbands for at least one reason. The more ICTs the households had, the less likely they were to justify wife beating for at least one reason. Women who listened to the radio less than once a week were less likely to justify wife beating than women who never listened to the radio. Women who watched television at least once a week were less likely to justify wife beating compared to women who never watched television. Women who read newspapers or magazines were associated with decreased odds of justifying wife beating than women who never read newspapers or magazines.

Furthermore, women aged 20–24 were associated with decreased odds of justifying wife beating, while women aged 35–39 and 45–49 were associated with increased odds of justifying wife beating compared to women aged 15–19. Higher levels of education and wealth were associated with decreased odds of justifying wife beating. Hindu women were less likely to justify wife beating compared to Muslim women, while rural women were more likely to justify wife beating compared to urban women. Women who were formerly in a union or living with a man were associated with decreased odds of justifying wife beating compared to women currently in a union/living with a man.

### 3.2. Multivariable Analysis

Multivariate logistic regression analysis was used to ascertain which ICTs were independently associated with women’s justification of wife beating for at least one reason. In Table 3, model 1 presented the AOR of household ownership of a mobile telephone, radio, television, and computer. In contrast, model 2 presented the AOR of the number of ICTs instead of specific ICTs, keeping other variables the same in both models. After potential confounding factors (i.e., frequency of reading newspapers or magazines, age, wealth, education, religion, and type of place of residence) were controlled, household ownership of a computer was found to be significantly associated with women’s justification for wife beating. Women with a computer in the household were less likely to justify wife beating than those without a computer (AOR = 0.73; 95% CI 0.57, 0.95). Women who had three ICTs in the household were associated with decreased odds of justifying wife beating compared to women who had no ICTs in the household (AOR = 0.67; 95% CI 0.47, 0.96). Furthermore, women who watched television at least once a week were less likely to justify wife beating compared to women who never watched television (model 1: AOR = 0.85; 95% CI 0.74, 0.97; model 2: AOR = 0.84; 95% CI 0.74, 0.97). The higher levels of education were associated with decreased odds of justifying wife beating (model 1: AOR = 0.36; 95% CI 0.29, 0.45; model 2: AOR = 0.36; 95% CI 0.29, 0.44). Additionally, Hindu women were less likely to justify wife beating compared to Muslim women (model 1: AOR = 0.54; 95% CI 0.42, 0.68; model 2: AOR = 0.54; 95% CI 0.42, 0.68).

## 4. Discussion

This study provides an insight into the association of the household ownership of ICTS (i.e., mobile telephones, radios, televisions, and computers), frequency of listening to radio, and frequency of watching television with women’s justifications for wife beating for at least one reason after adjustment for potential confounders, such as frequency of reading a newspaper or magazine, age, wealth, education, religion, and type of place of residence (see Table 3). The independent predictors were household ownership of ICTs (i.e., mobile telephones, radios, televisions, and computers), frequency of listening to radio, and frequency of watching television. Women’s approval or justification of wife beating for at least one reason of the five reasons (i.e., if the wife burns the food; if the wife neglects the children; if the wife argues with the husband; if the wife goes out without telling the husband; and if the wife refuses to have sex with the husband) are the outcome variable. Of the 20,032 women in this study, 3901 (19.47%) women approved or justified wife beating for at least one reason.

Household ownership of ICTs (i.e., mobile telephone, radio, television, and computer) is a predictor of justification for wife beating. In this study, the household ownership of a computer (AOR = 0.73 [95% CI = 0.57, 0.95]) was associated with decreased odds of justifying wife beating for at least one reason after the adjustment for the frequency of reading a newspaper or magazine, age, wealth, education, religion, and type of place of residence. One plausible reason for the effect of computers is that women can access awareness programs and resources on violence against women using this technology. The other reason is that the ownership of computers is related to socio-economic class. In other words, households that had computers should be more affluent than those who had no computers, which may already affect women’s awareness about violence against women. In contrast, household ownership of television had no significant association with justification for wife beating. These results were similar to the results of Cardoso and Sorenson [35] while dissimilar to the findings of Jensen and Oster [48]. Jensen and Oster [48] reported that household ownership of cable television was associated with rural Indian women’s lower likelihood to accepting wife beating. Moreover, we found no significant association between household ownership of mobile telephones and radio with IPV, which is inconsistent with an earlier study [35] which found a significant association between household ownership of mobile telephones and radios with IPV. This finding confirms that not all ICTs contribute equally in reducing IPV. There might be some reasons for this variation. One plausible reason may be the high costs of buying and accessing information technologies. This problem leads women victimized by IPV to a digital disparity in both high-income and low- and middle-income countries [34]. This disparity is a significant issue in electronic health (eHealth) [61,62]. Thus, digital disparity may influence the outcomes related to the effectiveness of ICTs in changing gender norms. Another reason is that ICT-based treatments only benefit women with basic literacy and IT skills [34]. In addition, traditional security issues such as the maintenance of privacy [63] and confidentiality [64], as well as ethical issues such as safety risks, are associated with the unintended consequences of ICT [65].

Apart from the individual relationships of ICTs, this study estimated the association of the number of ICTs with wife beating justifications. In this study, women in households with 3 ICTs (AOR = 0.67 [95% CI = 0.47, 0.96]) were associated with decreased odds of justifying wife beating for at least one reason. This finding is partially consistent with Cardoso and Sorenson [35], who found that among women residing in households with one to five ICTs, the likelihood of rejecting reasons for wife beating was higher than those in households without ICTs.

Behavioral characteristics such as listening to the radio and watching television were found to be the predictor of wife beating justifications. This study has observed that women who watched television at least once a week (AOR = 0.85 [95% CI = 0.74, 0.97]) were less likely to justify wife beating for at least one reason than women who never watched television. Similar findings have been revealed in Ghana [39]. One explanation of this result is that television is associated with changing gender norms [66,67,68]. The result indicates that despite the insignificant role of having ICTs in the household (in our case, television), the frequency of using ICTs (in our case, frequency of watching television) can have a significant association with intimate partner violence. Therefore, if we want to obtain the maximum benefit from an ICT, we must ensure that the target population uses that ICT. In contrast, Dickson et al. [39] found a significant association between the frequency of listening to the radio as well as the frequency of reading newspapers or magazines and the justification of wife beating, while this study found no such significant association.

This study found that the higher the level of educational attainment (e.g., incomplete secondary: AOR = 0.73 [95% CI = 0.63, 0.83]; complete secondary: AOR = 0.48 [95% CI = 0.36, 0.65]; higher: AOR = 0.36 [95% CI = 0.29, 0.45]), the less likely women are to justify wife beating than women who had no education. This result is consistent with previous literature [39,40,41,42,43,44,45,46,47]. Education is a key factor for many reasons, such as it fosters economic independence [10], which can empower women to deal with a controlling partner; it is the most influential demographic factor in determining attitudes towards wife beating [69]; and it helps to rely on logical reasoning and shares understanding rooted in contemporary and rational thinking [44,70]. Moreover, Hindu women (AOR = 0.54 [95% CI = 0.42, 0.68]) were less likely to justify wife beating compared to Muslim women. Previous research indicates that Muslim women are more accepting of wife beating than other religious followers like Christians [10] and Orthodox women [49]. This association is due to the influence of religious customs, teachings, fundamental principles, religious groups, and institutions that transmit values and belief systems to their adherents [49,50,51,52,53], as well as the influence of conservative and conventional beliefs [71].

### 4.1. Implications for Policy, Practice, and Future Research

Addressing the public health issues of the Bangladeshi residents is of great importance to the Bangladesh government’s policy. The first goal of the National Health Policy-2011 of Bangladesh is to provide fundamental health services among people of all social strata to improve public health and establish health as a right by Article 18(1) of the Constitution and other international conventions [72]. Additionally, the tenth goal of the National Health Policy-2011 of Bangladesh is to ensure the maximum and best use of information technology for comprehensive health sector management, including health services [72]. Ensuring gender equality in healthcare is the ninth goal of the policy and the fifth goal of the Sustainable Development Goals [73]. Furthermore, the WHO [74] has observed that Africa, South-Eastern Asia, and the Eastern Mediterranean demonstrate the highest incidence of physical domestic violence. The incidence of violence is high among women in the reproductive age range (15–49 years), which is a crucial time for maternal health [75,76]. Thus, some measures have been proposed to reduce this occurrence, such as the Declaration on the Elimination of Violence Against Women (DEVAW) and the UN Special Rapporteur on Violence Against Women [39]. This study result has implications for these policies and contexts. We discussed above how IPV is a public health concern associated with ICT.

We found that the frequency of watching television was associated with women’s justification of wife beating; policymakers can leverage this ICT to broadcast awareness programs designed for women and align the programs with their leisure time. Moreover, those responsible for television content should be aware of the choice of content that may negatively influence women. Additionally, since household ownership of computers was associated with women’s justification for wife beating, the government can help households buy computers.

ICTs, such as household ownership of mobile telephones and radios, were not significantly associated with women’s rejection of wife beating justifications. We need further research to investigate how Bangladeshi women use these ICTs, whether there is any gender inequality in assessing these ICTs among women experiencing IPV, and whether women who are experiencing IPV can use these ICTs to be conscious of harmful gender norms. In addition, this study’s findings imply a need to increase the use of information technology for health services among women experiencing IPV. Further research can also explore how Bangladeshi women use mobile telephones, whom they talk to, and whether they discuss gender norms over the phone, leveraging qualitative methods. Are there any fundamental differences between various ICTs’ contents, and how are these contents associated with women’s gender norms related to IPV? In addition, researchers can explore what programs or social media platforms the respondents are using; for instance, is it Bollywood/Hollywood movies that raise awareness on violence against women or YouTube/TikTok videos? Further studies can explore whether women use computers to learn about their rights or only use them for everyday work.

Since the current study only investigates women’s justifications towards IPV, future studies can examine whether ownership of information technology by the husband (and men generally) is negatively associated with justifications for IPV, especially if they have exposure to violent pornography or other types of programs that promote violence against women. Further research can also explore how the government can address this issue. Moreover, future studies may investigate the violence against women perpetrated by the mother-in-law or by the husband with the support of the mother-in-law.

### 4.2. Strengths and Limitations

An essential strength of this study is that it employed nationally representative data with a substantial sample size, enabling generalizations of the findings at the national level. Additionally, this study explored various factors that are associated with IPV. Understanding the association is important because the prevalence of IPV and women’s permissive attitudes towards wife beating are high in Bangladesh [43]. This study’s findings will help the government and stakeholders reduce IPV in Bangladesh.

The significant limitations of this study are related to its data. Earlier studies mentioned the limitations of using BDHS data [38]. The primary limitation of this study pertains to the cross-sectional design of the data, which hinders the establishment of a causal link. Additionally, the data utilized in this study were derived from self-reported assessments, which may be susceptible to potential social and cultural biases. Furthermore, the measurement of women’s attitudes towards IPV in the BDHS was conducted using a questionnaire consisting of five items. However, it is worth noting that this questionnaire did not encompass a comprehensive range of potential questions [77], which could have provided a more comprehensive understanding of their approval of IPV. The BDHS 2017-18 just gathered demographic and socio-economic information, excluding crucial factors about IPV, such as the length of marriage and the number of marriages.

## 5. Conclusions

This study examines the association between household ownership of ICTs (mobile telephones, radios, televisions, and computers), the frequency of listening to the radio, the frequency of watching television, and women’s justifications for wife beating. This study found that 3901 (19.47%) of the total 20,032 women approved or justified wife beating for at least one reason. The household ownership of computers was associated with decreased odds of justifying wife beating after adjusting for factors such as reading newspapers or magazines, age, wealth, education, religion, and type of place of residence. The study also found that women in households with three ICTs were associated with decreased odds of justifying wife beating for at least one reason. Behavioral characteristics like the frequency of watching television were associated with wife beating justifications. Women with higher educational levels (i.e., incomplete secondary, complete secondary, and higher) were less likely to justify wife beating than women without education. Religious beliefs also played a role in women’s approval/justification of wife beating. Hindu women were less likely to approve/justify wife beating compared to Muslim women. Since being tolerant of IPV has positive impacts on its perpetration [78], identifying the factors beyond the tolerance of IPV is essential. In this regard, this study has contributed to the literature by assessing the factors associated with the acceptance of IPV.

## Figures and Tables

**Table 1:** Household ownership of information and communication technologies (ICTs), frequency of listening to radio, frequency of watching television, and socio-demographic characteristics of participants (*N* = 20,032 *).

Characteristic	Number (*n*)	Percentage (%)
Household has mobile telephone		
No	641	3.20
Yes	19,391	96.8
Household has radio		
No	19,824	98.96
Yes	208	1.04
Household has television		
No	9926	49.55
Yes	10,106	50.45
Household has computer		
No	18,629	93.00
Yes	1403	7.00
Number of ICTs		
0	490	2.45
1	9337	46.61
2	8875	44.30
3	1299	6.48
4	31	0.15
Frequency of listening to radio		
Not at all	19,053	95.12
Less than once a week	555	2.77
At least once a week	423	2.11
Frequency of watching television		
Not at all	7331	36.60
Less than once a week	1757	8.77
At least once a week	10,944	54.63
Frequency of reading newspaper or magazine		
Not at all	17,917	89.44
Less than once a week	1315	6.56
At least once a week	800	3.99
Age in 5-year groups		
15–19	1935	9.66
20–24	3498	17.46
25–29	3554	17.74
30–34	3449	17.22
35–39	2937	14.66
40–44	2320	11.58
45–49	2339	11.68
Wealth index combined		
Poorest	3805	18.99
Poorer	3816	19.05
Middle	3868	19.31
Richer	4073	20.33
Richest	4470	22.31
Education		
No education	3181	15.88
Incomplete primary	4205	20.99
Complete primary	2111	10.54
Incomplete secondary	6877	34.33
Complete secondary	841	4.20
Higher	2817	14.06
Religion		
Islam	18,047	90.09
Hinduism	1855	9.26
Buddhism	84	0.42
Christianity	46	0.23
Type of place of residence		
Urban	7345	36.67
Rural	12,687	63.33
Marital status		
Currently in union/living with a man	18,815	93.92
Formerly in union/living with a man	1217	6.08
Sex of head of household		
Male	17,193	85.83
Female	2839	14.17

* Among the variables shown in the table, only ‘frequency of listening to radio’ has 20,031 observations.

**Table 2:** Association between household ownership of ICTs, frequency of listening to radio, frequency of watching television, as well as socio-demographic characteristics and women’s justifications for wife beating for at least one reason (*N* = 20,032 ^#^).

Variable	Women Agree That Husband Is Justified in Hitting or Beating His Wife for at Least One Reason		Univariable Odds Ratio ^a^	
	No *n* (%)	Yes *n* (%)	OR	95% CI
Household ownership of ICTs				
Household has mobile telephone				
No	480 (74.88)	161 (25.12)	1.00	
Yes	15,651 (80.71)	3740 (19.29)	0.74	0.59, 0.93 *
Household has radio				
No	15,962 (80.52)	3862 (19.48)	1.00	
Yes	169 (81.25)	39 (18.75)	0.91	0.59, 1.40
Household has television				
No	7657 (77.14)	2269 (22.86)	1.00	
Yes	8474 (83.85)	1632 (16.15)	0.72	0.64, 0.80 **
Household has computer				
No	14,858 (79.76)	3771 (20.24)	1.00	
Yes	1273 (90.73)	130 (9.27)	0.45	0.35, 0.57 **
Number of ICTs				
0	356 (72.65)	134 (27.35)	1.00	
1	7217 (77.29)	2120 (22.71)	0.77	0.60, 0.97 *
2	7353 (82.85)	1522 (17.15)	0.59	0.46, 0.76 **
3	1176 (90.53)	123 (9.47)	0.31	0.23, 0.43 **
4	29 (93.55)	2 (6.45)	0.23	0.06, 0.89 *
Frequency of listening to radio				
0	15,288 (80.24)	3765 (19.76)	1.00	
<1 per week	484 (87.21)	71 (12.79)	0.64	0.48, 0.84 *
≥1 per week	359 (84.87)	64 (15.13)	0.82	0.59, 1.13
Frequency of watching television				
0	5566 (75.92)	1765 (24.08)	1.00	
<1 per week	1403 (79.85)	354 (20.15)	0.85	0.71, 1.01
≥1 per week	9162 (83.72)	1782 (16.28)	0.68	0.60, 0.76 **
Frequency of reading newspaper or magazine				
0	14,236 (79.46)	3681 (20.54)	1.00	
<1 per week	1162 (88.37)	153 (11.63)	0.54	0.44, 0.66 **
≥1 per week	733 (91.62)	67 (8.38)	0.38	0.27, 0.54 **
Age (years)				
15–19	1562 (80.72)	373 (19.28)	1.00	
20–24	2915 (83.33)	583 (16.67)	0.85	0.73, 0.99 *
25–29	2912 (81.94)	642 (18.06)	0.97	0.84, 1.12
30–34	2781 (80.63)	668 (19.37)	1.05	0.90, 1.22
35–39	2311 (78.69)	626 (21.31)	1.18	1.01, 1.37 *
40–44	1834 (79.05)	486 (20.95)	1.08	0.91, 1.28
45–49	1816 (77.64)	523 (22.36)	1.21	1.04, 1.42 *
Wealth index				
Poorest	2854 (75.01)	951 (24.99)	1.00	
Poorer	2964 (77.67)	852 (22.33)	0.89	0.78, 1.02
Middle	3074 (79.47)	794 (20.53)	0.81	0.69, 0.96 *
Richer	3329 (81.73)	744 (18.27)	0.74	0.62, 0.88 *
Richest	3910 (87.47)	560 (12.53)	0.50	0.42, 0.61 **
Education				
No education	2340 (73.56)	841 (26.44)	1.00	
Incomplete primary	3203 (76.17)	1002 (23.83)	0.91	0.81, 1.03
Complete primary	1644 (77.88)	467 (22.12)	0.86	0.74, 1.00 *
Incomplete secondary	5620 (81.72)	1257 (18.28)	0.67	0.60, 0.76 **
Complete secondary	741 (88.11)	100 (11.89)	0.42	0.32, 0.55 **
Higher	2583 (91.69)	234 (8.31)	0.28	0.23, 0.34 **
Religion				
Islam	14,397 (79.78)	3650 (20.22)	1.00	
Hinduism	1635 (88.14)	220 (11.86)	0.53	0.43, 0.67 **
Buddhism	61 (72.62)	23 (27.38)	1.59	0.76, 3.33
Christianity	38 (82.61)	8 (17.39)	0.99	0.28, 3.47
Type of place of residence				
Urban	6153 (83.77)	1192 (16.23)	1.00	
Rural	9978 (78.65)	2709 (21.35)	1.36	1.14, 1.62 **
Marital status				
Currently in union/living with a man	15,127 (80.40)	3688 (19.60)	1.00	
Formerly in union/living with a man	1004 (82.50)	213 (17.50)	0.83	0.70, 0.99 *
Sex of head of household				
Male	13,835 (80.47)	3358 (19.53)	1.00	
Female	2296 (80.87)	543 (19.13)	0.98	0.86, 1.11

*OR* Odds ratio, *95%CI* 95% confidence interval. ^a^ The univariable odds ratio indicates the likelihood of women’s justifications for wife beating for at least one reason * *p* < 0.05 ** *p* < 0.001 ^#^ Among the variables shown in the table, only ‘frequency of listening to radio’ has 20,031 observations.

**Table 3:** Adjusted odds ratios (AORs) ^c^ for association between household ownership of ICTs, frequency of listening to radio, frequency of watching television and women’s justifications for wife beating for at least one reason after adjustment for potential confounders ^b^ (*n* = 20,031).

Variable ^a^	Model 1			Model 2		
	AOR	95% CI	*p*-Value	AOR	95%CI	*p*-Value
Household ownership of ICTs						
Mobile telephone						
No	1.00					
Yes	0.92	0.73, 1.17	0.501			
Radio						
No	1.00					
Yes	1.10	0.69, 1.76	0.684			
Television						
No	1.00					
Yes	1.01	0.87, 1.16	0.939			
Computer						
No	1.00					
Yes	0.73	0.57, 0.95	0.018			
Number of ICTs						
0				1.00		
1				0.85	0.66, 1.09	0.197
2				0.87	0.65, 1.17	0.353
3				0.67	0.47, 0.96	0.028
4				0.54	0.13, 2.17	0.386
Frequency of listening to radio						
Not at all	1.00			1.00		
Less than once a week	0.90	0.68, 1.21	0.493	0.91	0.68, 1.22	0.525
At least once a week	1.18	0.83, 1.67	0.368	1.20	0.85, 1.70	0.292
Frequency of watching television						
Not at all	1.00			1.00		
Less than once a week	0.90	0.76, 1.08	0.264	0.90	0.76, 1.08	0.255
At least once a week	0.85	0.74, 0.97	0.018	0.84	0.74, 0.97	0.013
Frequency of reading newspaper or magazine						
Not at all	1.00			1.00		
Less than once a week	0.88	0.71, 1.08	0.223	0.88	0.71, 1.08	0.211
At least once a week	0.85	0.59, 1.22	0.364	0.84	0.59, 1.21	0.360
Age in 5-year groups						
15–19	1.00			1.00		
20–24	0.91	0.78, 1.05	0.681	0.91	0.78, 1.06	0.216
25–29	0.97	0.83, 1.13	0.681	0.97	0.83, 1.13	0.697
30–34	0.99	0.84, 1.16	0.865	0.99	0.84, 1.16	0.893
35–39	1.04	0.88, 1.23	0.625	1.04	0.89, 1.23	0.619
40–44	0.89	0.74, 1.08	0.246	0.89	0.74, 1.08	0.246
45–49	0.98	0.83, 1.16	0.817	0.98	0.82, 1.16	0.796
Wealth index combined						
Poorest	1.00			1.00		
Poorer	0.96	0.84, 1.10	0.581	0.97	0.84, 1.11	0.624
Middle	0.98	0.83, 1.16	0.824	0.98	0.83, 1.16	0.809
Richer	1.01	0.84, 1.21	0.951	1.00	0.84, 1.21	0.962
Richest	0.91	0.72, 1.15	0.411	0.89	0.71, 1.13	0.348
Educational attainment						
No education	1.00			1.00		
Incomplete primary	0.93	0.82, 1.05	0.240	0.93	0.82, 1.05	0.242
Complete primary	0.88	0.76, 1.03	0.117	0.88	0.76, 1.03	0.117
Incomplete secondary	0.73	0.63, 0.83	<0.001	0.73	0.63, 0.83	<0.001
Complete secondary	0.48	0.36, 0.65	<0.001	0.48	0.36, 0.65	<0.001
Higher	0.36	0.29, 0.45	<0.001	0.36	0.29, 0.44	<0.001
Religion						
Islam	1.00			1.00		
Hinduism	0.54	0.42, 0.68	<0.001	0.54	0.42, 0.68	<0.001
Buddhism	1.64	0.89, 3.01	0.110	1.63	0.89, 2.96	0.112
Christianity	1.07	0.34, 3.36	0.903	1.06	0.34, 3.33	0.916
Type of place of residence						
Urban	1.00			1.00		
Rural	1.17	0.96, 1.41	0.111	1.17	0.96, 1.41	0.111

^a^ All variables in the final model were variables for which, when excluded, the change in deviance compared with the corresponding χ^2^ test statistic on the relevant degrees of freedom was significant. ^b^ Adjusted for frequency of reading newspaper or magazine, age, wealth, education, religion, and type of place of residence. ^c^ *AOR* adjusted odds ratio, *95%CI* 95% confidence interval.

## Data Availability

Data associated with this study is available at https://dhsprogram.com/data/Using-Datasets-for-Analysis.cfm (accessed on 24 October 2024).

## Abbreviations

ICTs	Information and Communication Technologies
IPV	Intimate Partner Violence
BDHS	Bangladesh Demographic and Health Survey
WHO	World Health Organization
LMICs	Low- and Middle-Income Countries
PTSD	Post-Traumatic Stress Disorder
ITU	International Telecommunication Union
MCI	Mobile Connectivity Index
NIPORT	National Institute of Population Research and Training
USAID	United States Agency for International Development
BBS	Bangladesh Bureau of Statistics
EAs	Enumeration Areas
PSU	Primary Sampling Unit
AOR	Adjusted Odds Ratio
OR	Odds Ratio
VIF	Variance Inflation Factor
DEVAW	Declaration on the Elimination of Violence Against Women

## Appendix A. Multicollinearity Diagnosis Result

**Table A1:** Multicollinearity diagnosis result.

Variable	VIF	1/VIF
Household ownership of ICTs		
Household has mobile telephone (vs. No)	1.05	0.955374
Household has radio (vs. No)	1.04	0.963669
Household has television (vs. No)	2.66	0.375782
Household has computer (vs. No)	1.27	0.788774
Frequency of listening to radio (vs. Not at all)		
Less than once a week	1.07	0.935759
At least once a week	1.06	0.942148
Frequency of watching Television (vs. Not at all)		
Less than once a week	1.18	0.850284
At least once a week	2.50	0.400086
Frequency of reading newspaper or magazine (vs. Not at all)		
Less than once a week	1.18	0.848031
At least once a week	1.28	0.782455
Age in 5-year groups (vs. 15–19)		
20–24	2.34	0.427214
25–29	2.38	0.420883
30–34	2.39	0.418738
35–39	2.31	0.432906
40–44	2.17	0.461448
45–49	2.27	0.441120
Wealth index combined (vs. Poorest)		
Poorer	1.74	0.573367
Middle	2.07	0.482707
Richer	2.42	0.412436
Richest	3.52	0.284487
Educational attainment (vs. No education)		
Incomplete primary	1.93	0.518150
Complete primary	1.61	0.621703
Incomplete secondary	2.73	0.366203
Complete secondary	1.40	0.715765
Higher	2.55	0.391409
Religion (vs. Islam)		
Hinduism	1.02	0.985110
Buddhism	1.00	0.996669
Christianity	1.00	0.997253
Type of place of residence (vs. Urban)		
Rural	1.29	0.772832
Mean VIF	1.81	
